# Identifying differential transcription factor binding in ChIP-seq

**DOI:** 10.3389/fgene.2015.00169

**Published:** 2015-04-29

**Authors:** Dai-Ying Wu, Danielle Bittencourt, Michael R. Stallcup, Kimberly D. Siegmund

**Affiliations:** ^1^Department of Biochemistry and Molecular Biology, University of Southern California Norris Comprehensive Cancer Center, University of Southern CaliforniaLos Angeles, CA, USA; ^2^Department of Preventive Medicine, University of Southern California Norris Comprehensive Cancer Center, University of Southern CaliforniaLos Angeles, CA, USA

**Keywords:** ChIP-seq, differential binding, methods comparison, normalization, validation

## Abstract

ChIP seq is a widely used assay to measure genome-wide protein binding. The decrease in costs associated with sequencing has led to a rise in the number of studies that investigate protein binding across treatment conditions or cell lines. In addition to the identification of binding sites, new studies evaluate the variation in protein binding between conditions. A number of approaches to study differential transcription factor binding have recently been developed. Several of these methods build upon established methods from RNA-seq to quantify differences in read counts. We compare how these new approaches perform on different data sets from the ENCODE project to illustrate the impact of data processing pipelines under different study designs. The performance of normalization methods for differential ChIP-seq depends strongly on the variation in total amount of protein bound between conditions, with total read count outperforming effective library size, or variants thereof, when a large variation in binding was studied. Use of input subtraction to correct for non-specific binding showed a relatively modest impact on the number of differential peaks found and the fold change accuracy to biological validation, however a larger impact might be expected for samples with more extreme copy number variations between them. Still, it did identify a small subset of novel differential regions while excluding some differential peaks in regions with high background signal. These results highlight proper scaling for between-sample data normalization as critical for differential transcription factor binding analysis and suggest bioinformaticians need to know about the variation in level of total protein binding between conditions to select the best analysis method. At the same time, validation using fold-change estimates from qRT-PCR suggests there is still room for further method improvement.

## Introduction

Chromatin immunoprecipitation combined with sequencing (ChIP-seq) is a technique used to identify DNA binding sites for proteins or histone modification of nucleosomes (Pepke et al., 2009; Furey, 2012). ChIP-seq experiments have become increasingly popular as sequencing costs decrease and more validated histone and transcription factor antibodies are available. Comparisons between ChIP-seq experiments can provide novel insight into differences in protein occupancy and histone marks (Xu et al., 2008; Wu and Ji, 2010; Ross-Innes et al., 2012; Shao et al., 2012; Ji et al., 2013; Wong et al., 2015). Since transcription factor binding signals often form narrow peaks of relatively uniform shape, differential count methods from RNA-seq, using peaks instead of genes, seem well-suited for data analysis. We review differential transcription factor (TF) binding methods for ChIP-seq (Xu et al., 2008; Song and Smith, 2011; Stark and Brown, 2011; Bardet et al., 2012; Liang and Keles, 2012; Nair et al., 2012; Shao et al., 2012), and compare the performance of these methods on several ENCODE data sets.

**Figure 1:**
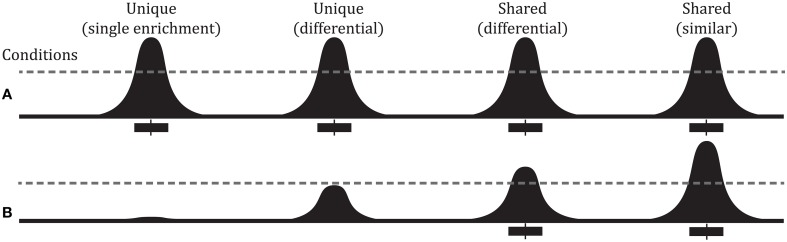
**Overview of peak types and defining reference binding regions. (A)** Several different types of peak comparisons are shown. The black curve represents binding signal with the dotted line representing a hypothetical threshold for enrichment. The black boxes under each curve represent significant regions as defined by peak caller output with the vertical line in the box representing summit point. Comparing binding profiles in conditions **(A)** vs. **(B)** we find: binding in condition **(A)** but not **(B)** (Unique—single enrichment), varying degrees of binding between the two conditions (Unique and Shared peak—differential), and both conditions having a peak of about comparable signal intensity (Shared peak—similar).

**Figure 2:**
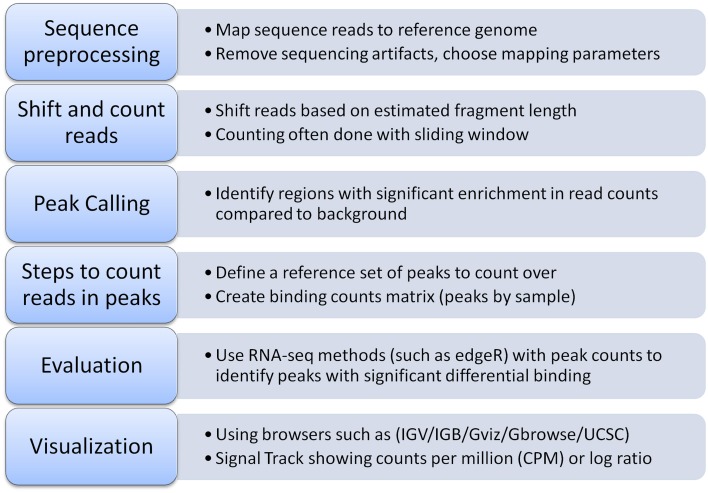
**Typical workflow for identifying differential transcription factor binding**.

Pairwise comparisons between ChIP-seq experiments can identify differential binding sites (Figure 1). The simplest way to identify differential peaks is by overlapping peak regions between conditions and classifying peaks as unique to one condition or shared by (common to) both conditions. Another approach is a quantitative comparison between conditions of the number of sequencing reads overlapping a peak (peak height). Such comparisons can identify differential binding in peaks shared by two conditions that could not be identified from a simple overlap of peak regions (Figure 1). RNA-seq has several methods to compare read counts between treatment conditions for an annotated feature set. For differential binding analysis, peak regions are the features of interest and are typically obtained from the ChIP-seq experiments. Figure 2 outlines a typical workflow to perform differential binding analysis [see Landt et al. (2012) for guidelines and (Park, 2009; Bailey et al., 2013) for review on ChIP-seq analysis].

A number of software tools are available to analyze differential TF binding. We review and evaluate methods adapted from differential RNA-seq that have variations to address issues specific to ChIP-seq data (Table 1). The methods are run on six high quality protein ChIP-seq datasets from the ENCODE project (Dunham et al., 2012). We use Glucocorticoid receptor (GR/NR3C1) and Estrogen receptor alpha (ERα/ESR1) data to assess increases in binding in response to hormone within a single cell type, TCF7L2 (TCF4) and NRF1 to assess cell-type specific binding, and use replicate experiments of Pol2 (POLR2A) and c-Myc (MYC) as negative controls. We expect increasing differential binding in GR and ERα hormone-response studies, some cell-type specific differential binding in TCF7L2 and NRF1 studies, and no differential binding in Pol2 and c-Myc negative controls.

**Table 1:** **Differential binding methods**.

**Method**	**Steps before counting reads in peaks**	**Evaluation**
Non-overlap	n/a	Not overlap 1bp+
edgeR	Shift reads	edgeR
DiffBind	Extend reads, scale down background	edgeR w/TMM w/bg^*^ subtract
MAnorm3	Shift reads, normalize using shared peaks (calculate MA adjustment)	edgeR + MA adjustment
voom	Shift reads + voom transform	eBayes

* *Background*.

We demonstrate that analysis step choices have a major impact on the results depending on the biological conditions investigated. From this, we highlight critical decisions and recommend analysis procedures for some standard biological study designs.

## Materials and methods

### Comparison of peak regions

The simplest way to compare binding between different biological conditions is to overlap peaks from different conditions to identify unique and shared peak regions. Determining the number of overlapping peaks involves defining a reference sample and counting overlapping peaks in other conditions. However, the number of peaks that overlap between two conditions can vary depending on choice of reference sample because a peak in one condition could overlap several peaks in another condition. Under these circumstances, the median number of overlaps from all pairwise comparisons is reported. A severe limitation of this approach is that the number of peaks found depends on sequencing depth, thus, overlap analysis can be severely biased when conditions are sequenced to different depths. We evaluate if methods that quantify differential binding can overcome this limitation.

### Comparison of binding levels in peak regions

Rather than overlapping peaks between conditions, a quantitative comparison of read counts within peak regions can be used to identify peaks with significantly different counts. RNA-seq methods are typically used to identify differences in counts over pre-defined features (genes). For ChIP-seq, features of interest (peaks) are defined from an analysis of the data and can vary between experiments. To perform differential TF binding, a reference set of binding regions is defined allowing results to be summarized in a matrix of counts. Different approaches for defining a reference set are available. At one extreme, the entire genome is binned into regions of fixed length. A more typical approach will simply merge overlapping peak regions from different experiments. Additionally, differential binding methods leverage characteristics related to ChIP-seq such as corrections for non-specific binding (“input”) and normalizing sequencing depth using shared peaks.

#### Effect of input

ChIP-seq experiments usually include input, a measure of non-specific background binding, as a negative control. Some factors that could contribute to background signal include copy number alterations, high mappability regions, or open chromatin. Some differential binding methods subtract input counts from binding counts to remove background signal. This requires an additional step to normalize input reads before subtraction. Since background does not vary when comparing treatment conditions within the same cell type this may not be recommended for all comparisons. Subtracting input lowers the counts, potentially reducing sensitivity to detect differential binding within cell type.

#### Normalization methods

Normalization is required to properly scale signal between different experiments. For sequencing, library size is an indicator of total signal. “Full” library size refers to the number of mapped reads in the sample and “effective” library size refers to the number of reads mapped to the features of interest. Although features of interest are not defined *a priori* for ChIP-seq analysis and Full library size might seem a natural choice to normalize samples for coverage, sometimes normalization is performed using only the counts for the features of interest (Liang and Keles, 2012; Ross-Innes et al., 2012), or a subset of the features (Shao et al., 2012) (reviewed by Bailey et al., 2013). A study of differential estrogen receptor binding (Ross-Innes et al., 2012) used TMM (trimmed mean of *M*-values) (Robinson and Oshlack, 2010), a method proposed for normalizing RNA-seq data when it can be assumed that most genes are not differentially expressed. The method trims the tails of the distribution of log fold-changes (*M*-values) prior to centering at zero. MAnorm (Shao et al., 2012), a normalization method designed specifically for differential protein binding, makes an assumption similar to no differential expression of most genes, assuming that most shared peaks are not differentially bound. It normalizes all counts based on a linear regression of log-fold change vs. average binding in shared peaks, peaks common to both experimental conditions. Table 2 summarizes the combinations of normalization methods that we evaluated. We show that the results, when comparing experiments with different levels of total protein bound, are very sensitive to the choice of normalization method. Our analysis allows us to suggest the best approach among those evaluated, and illustrate the many false positive and false negative results that can arise from a wrong analysis.

**Table 2:** **Summary of comparisons**.

	**Library size**				**Input subtraction**	
	**Effective**	**Full**	**TMM**	**MAnorm**	**None**	**scale CPM**
edgeR default	x		x		x	
edgeR full		x			x	
DiffBind default	x		x			x
DiffBind full		x	x			x
MAnorm3	x^*^			x	x	
Voom		x			x	

*Each type of analysis is shown with a different row with X indicating the option(s) that was chosen*.

* *MAnorm3 takes the average of log transformed effective library sizes for scaling*.

### Data sets and methods comparison

We use six TF datasets from ENCODE (mar 2012 data release) to evaluate different software for differential binding analysis. For each of the six studies, the ChIP-seq experiments are produced by the same lab, in replicates with multiple different biological conditions. Study designs we considered are: cell type-specific differences and treatment differences within a cell type. In the first design, the same protein was assayed in different cell types to discover differential binding between cell type (TCF7L2, NRF1); in the second, the same protein was assayed across multiple conditions in the same cell line to look for treatment-related effects (GR, ERα). Lastly, we analyzed two negative control data sets with the same protein binding experiment repeated multiple times in the same cells lines (Pol2/c-Myc). The sequence data were single-end reads, 27–36 base pairs in length depending on the experiment, and performed on Illumina sequencers. Two groups produced the data, Hudson Alpha (HAIB) (GR, ERα) and Snyder's group (Sydh) (Pol2, c-Myc, TCF7L2, NRF1). Evaluation of a subset of bam files from each group indicated that Sydh samples had mean quality values ~40 while HAIB samples had mean quality values ~30. The advantages of using ENCODE datasets include the high quality standard for biological experiments, the availability of replicates, multiple experiments from the same lab, and consistent processing of data for mapping and peak calling. Peaks were generated from a modified SPP pipeline (Anshul Kundaje, Lucy Yungsook et al. Assessment of ChIP-seq data quality using cross-correlation analysis. Submitted) which incorporates the IDR framework (Li et al., 2011) to increase reproducibility. For reads, we used the tagAlign files that were converted from bam files and use less disk space. Peaks were IDR output with an additional filter *q* < 0.01 for peak detection. Both of these files are publicly available from ENCODE (See Supplemental Table 1 for file names and links to data).

We perform differential binding analysis with tools that utilize edgeR (version 3.0.8) (Robinson et al., 2010), an approach developed for RNA-seq and shown to perform well with small numbers of replicate samples (Rapaport et al., 2013). The four methods we compare include: (1) edgeR with either TMM or full library size normalization; (2) DiffBind (version v1.4.2) (Stark and Brown, 2011), which adds a step to scale input prior to performing differential analysis using edgeR. The same input samples that were used for peak calling are used for scaling; (3) a modified implementation of MAnorm (Shao et al., 2012) to allow for replicate ChIP-seq experiments in the normalization of samples across shared peak regions, and use edgeR for differential peak calling; and (4) Voom (version 3.14.4) (Law et al., 2014), a method that transforms Poisson-based read counts into normal-based signal values that can be used with pre-existing microarray analysis methods. With the exception of MAnorm, these methods can be found on Bioconductor (Gentleman et al., 2004). MAnorm was rewritten and will be referred to as MAnorm3, availability and list of major changes can be found in Supplemental Table 2.

All normalization procedures are implemented using an offset variable in the regression model for differential binding. MAnorm implicitly uses effective library size and Voom used full library size for transformation with limma (Smyth, 2004) for microarray differential expression analysis. Unless otherwise mentioned, all methods were performed with a false-discovery rate (FDR)-adjusted cutoff of *p* < 0.05.

Our analysis included heavy use of GenomicRanges package (version 1.10.7) (Lawrence et al., 2013). For each dataset, counts were obtained by using coverageBed (Quinlan and Hall, 2010) to count reads overlapping peaks. The reference set of peak regions used for the binding count matrix was obtained by merging peaks overlapping 1 bp from the ChIP experiments.

### ChIP-qPCR validation

We performed validation of fold changes via ChIP-qPCR for GR in A549 cell lines. We choose a mixture of regions that represented both shared and unique peaks. We followed the previously described protocol for antibody and cell growth conditions as well as treatment conditions (Reddy et al., 2012). Chromatin immunoprecipitation (ChIP) was performed as previously described (Bittencourt et al., 2012) except that cells were cross-linked for 10 min at room temperature with formaldehyde only. IP signals were normalized relative to the signal obtained from input chromatin and fold changes were calculated by dividing normalized IP signal values followed by log2 transformation. Up to three qPCR technical replicates were performed for each experiment along with IgG and 2% input chromatin as control.

## Results

### Peak region-based analysis

**Figure 3:**
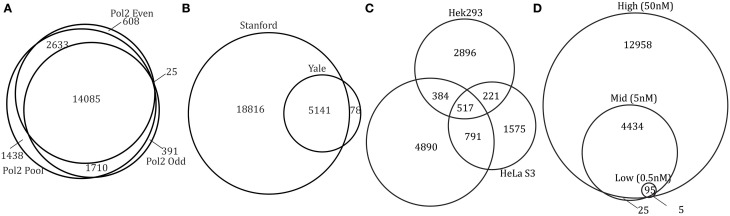
**Results from condition-level comparison methods**. Venn diagrams of overlapping peak regions (**A**, Pol2; **B**, c-Myc; **C**, TCF7L2; **D**, GR).

Overlapping the peak regions for two conditions is the simplest way to identify differential binding. Overlapping our Pol2 peaks from two subsets of the data (odd replicates and even replicates), we find that the two sets of peaks highly overlap (>80%). An even higher overlap is observed when comparing peaks from each subset to the peaks identified from the pool of reads from all six replicates (96–98% overlap with pooled) (Figure 3A). This result is expected since Pol2 is a negative control and we compare peaks from replicates of the same experimental condition.

Overlap analysis comparing experiments sequenced to different depths is subject to finding unique peaks due to sequencing depth alone. To illustrate the effect of sampling depth on peak reproducibility we created data sets with 8 M, 14.4 M, and 46.2 M reads from a combination of pooling and sub-sampling independent Pol2 replicates (replicates 1–4, replicates 5–6, and sub-sampling replicates 5–6). These three data sets show an increasing number of peaks with increasing read depth (8577, 15851, and 18287 peaks). An overlap analysis found 9059 (49.5%) unique peaks in the high depth sample that did not overlap any of the 8577 peaks discovered in the sample with five-fold lower sequencing depth. The number of unique peaks was halved for the smaller differential in sequencing depth (three-fold difference in coverage, 4374 (23.5%) unique peaks). In the c-Myc control experiment, replicate experiments were conducted by the same lab, but in two different institutions (first at Yale then at Stanford several years later). The more recent experiment used a different input control and was sequenced more deeply (~20 M reads per replicate vs. ~4 M reads per replicate). The overlap of peaks in Figure 3B shows that the more recent Stanford dataset has many more peaks than the Yale dataset, once more highlighting the importance of sequencing depth when identifying differential peaks using overlaps as criteria. To account for the extreme difference in the number of peaks found between the two c-Myc experiments, we filtered all but the top 5000 peaks (the approximate number found in the Yale data set) and find that over 70% of the peaks overlap. Filtering additional peaks did not increase the overlapping proportion above 75% (data not shown). The lower 75% overlap compared to the over 80% observed for Pol2 is not surprising given the length of time separating these two c-Myc experiments. This suggests that a few of the peaks with highest occupancy might have changed during the lag time between the two experiments.

Overlapping binding sites between cell types identifies both cell type-specific binding as well as shared binding sites between cell-types, a result that has been reported previously (Frietze et al., 2012). For TCF7L2, fewer peaks were identified in the HeLa S3 cell line and fewer cell type-specific peaks were identified in HeLa S3 (49%) compared to HEK293 (72%) and MCF7 (74%) (Figure 3C). In NRF1 experiments, much less cell type-specific binding sites are found with over 60% of peaks shared by all three cell types (GM12878, H1 hESC, K562) and fewer than 20% unique to each cell type (Supplemental Figure 1).

Overlapping GR peaks from different hormone concentrations (Figure 3D), we find GR binding sites increases with hormone concentration, a result consistent with biology since GR requires hormone to bind DNA. Almost all peaks found in lower hormone concentration are a subset of peaks from higher hormone treatment. All 25 regions specific to medium hormone binding either have very low enrichment or have a peak in high hormone treatment nearby. In ERα datasets, treatment with three different types of hormones (bpa, genistein, and estradiol) caused differential binding with bpa-specific binding sites being a subset of genistein and estradiol binding sites (Supplemental Figure 1). This finding supports the conclusion from the ERα datasets that bpa and genistein induce a subset of estradiol treatment effects (Gertz et al., 2012).

In conclusion, overlapping peaks from different conditions is sensitive to sequencing depth. The number of peaks found at the same FDR cutoff will change if the samples being compared have large differences in sequencing depth.

### Binding level change analysis

#### edgeR

**Figure 4:**
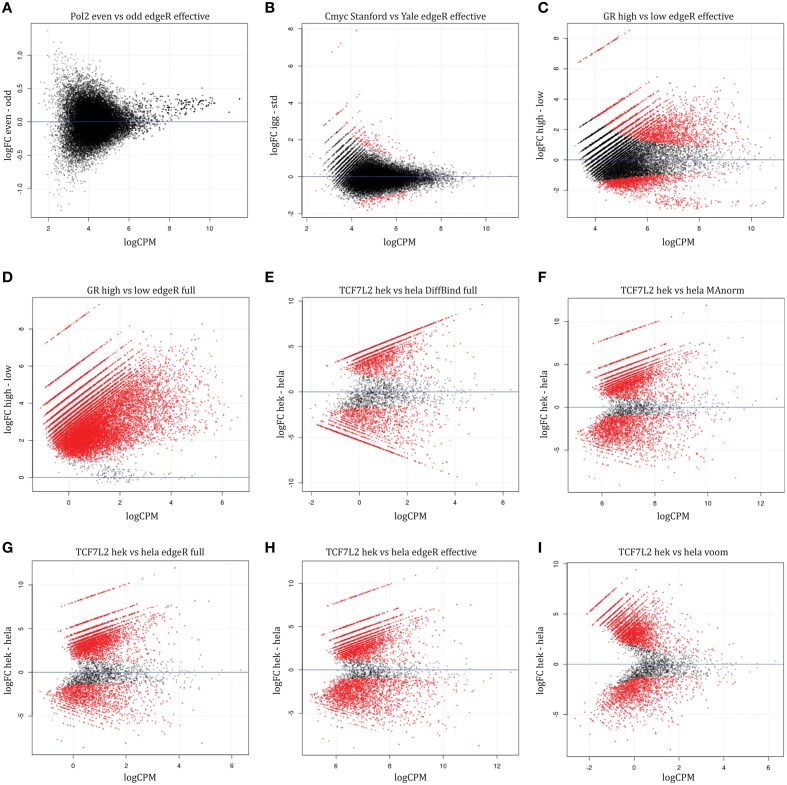
**MA plots for sample-level comparison methods**. Regions with significant differential binding are highlighted in red. Controls: **(A)** Pol2 comparison, **(B)** c-Myc comparison. Normalization differences: GR comparison between high vs. low hormone using **(C)** effective or **(D)** full library size normalization. TCF7L2 comparison between HEK293 and Hela S3 cells using **(E)** DiffBind with full library size, **(F)** MAnorm3, **(G)** edgeR with full library size, **(H)** edgeR with effective library size, **(I)** voom.

We apply the RNA-seq method edgeR to read counts from ChIP-seq to identify differential binding using a reference set of peak regions (see Materials and Methods). *edgeR* utilizes negative binomial modeling, an approach demonstrated to have good specificity and sensitivity for differential expression, with good control of type I error (Rapaport et al., 2013). Two types of normalization are used with edgeR: effective library size (the default) will normalize to total number of reads overlapping features (genes/peaks) using TMM while full library size normalizes to the total number of reads in a sample. Normalizing using both full and effective library size, we found no differential peaks in Pol2 dataset (Figure 4A). Our other control, c-Myc, had 292 (about 1% of peaks) differentially bound regions using effective library size normalization, fewer than the 5% family-wise error rate used as cutoff, and vastly less than the number of differential regions found by the overlaps method. These differential regions can be seen in the MA plots (Figure 4B), plots of log-fold change (*M*-value on vertical axis) against average log counts (peak height, “A” on horizontal axis). These differential sites are not significant when normalizing using full library size (Table 3), suggesting that full library size is a more conservative adjustment for different sequencing depth.

**Table 3:** **Number of significant differential binding regions**.

	**Pol2 Odd vs. Even**	**c-Myc stanford vs. yale**	**TCF Hek293 vs. HelaS3**	**NRF1 Gm878 vs. H1esc**	**GR High vs. Low**	**ERa bpa vs. est**
Non-overlap	4885	17,962	5314	1497	17,339	15,730
edgeR efflib	0	292	5199	1687	4318	223
edgeR fulllib	0	0	4627	1738	17,246	10,986
DiffBind efflib	5	411	5238	1732	2908	9
DiffBind fulllib	46	7	4663	1594	17,233	9063
MAnorm3	0	1991	5063	1638	14,249	897
voom fulllib	0	1	4496	1206	17,215	10,914
Number of peaks	16,278	22,828	5976	4089	17,439	15,968

*This table shows the number of significantly differential binding sites for each of the methods where significant differential is defined as FDR adjusted p-value of less than 0.05 except for non-overlap where non-overlap is the sum of the unique sites*.

Normalizing for library size showed dramatic differences when assaying differential binding between hormone treatments. Many more differential GR and ERα binding sites are found when using full library size instead of effective library size (Figures 4C,D, Table 3, Supplemental Table 3, except for ER est vs. gen). Biologically, we expect most sites to be bound only in the high hormone treatment condition for GR. As hormone concentration decreases, a lower proportion of available binding sites will be occupied by GR with only the hypersensitive binding sites occupied at low hormone treatment. The MA plot from using full library size for normalization matches the biology for GR—most sites are differentially bound and higher fold changes when comparing high hormone treatment to low hormone treatment (Figure 4D). When using effective library size for normalization, a contradiction arises where we find significantly reduced binding in high hormone treatment compared to low hormone treatment (Figure 4C). Both datasets have similar sequencing depth but different amounts of protein bound, suggesting that normalization using full library size is more robust to variations in bound protein concentration than effective library size.

When comparing between different cell types (TCF7L2 and NRF1 experiments), we find that similar numbers of differentially bound sites with a high degree of overlap using the two different normalization methods (Table 3). Sequencing depth is roughly similar between the cell types and we assume that total protein binding is also similar. Under these two conditions, only a small difference between the two library size normalization methods is observed (Figures 4G,H), suggesting that the choice of library size has little effect on results when we do not expect varying protein binding between conditions and sequencing depth is similar. We conclude that using edgeR with full library size normalization performs similar to, or better than, edgeR with effective library size normalization at detecting differentially bound regions.

#### DiffBind

This method extends on edgeR by subtracting scaled input (background) read counts from read counts overlapping peaks. We expect DiffBind to perform similarly to edgeR for the control and hormone treatment datasets since they are performed on the same cell type. However, when comparing between different cell types, input subtraction might correct for differences in regional DNA copy number alterations (CNAs).

DiffBind performs similarly to edgeR when using full library size normalization, with similar or fewer number of differential peaks found by DiffBind in most comparisons (Table 3, Supplemental Table 3). When assessing GR binding in A549 cells, subtracting background has no effect on the number of differential loci found for large differences in peak height (high vs. low dose hormone treatment), but results in fewer differential loci for the more moderate differences (high vs. med dose). This is not surprising as there are no differences in copy number for within cell line comparisons, and larger differences can better tolerate input subtraction without loss of signal. Interestingly, using DiffBind yielded a similar number of differentially bound peaks between cell types compared to edgeR (no input subtraction) (Table 3, Figures 4E,G). Known CNAs exist for these different cell types and the proteins did bind in regions with CNAs (Supplemental Table 4). We speculated that correcting for input could increase true-positives at the same time it decreases false-positives in regions with copy number differences. We explored this using CNA summary data published by ENCODE (Supplemental Tables 4, 5).

CNAs between cell lines are difficult to quantify from the summary data, however assessing the enrichment of differential peaks in regions of amplification/deletion for different cell lines and analysis methods is informative. With or without input correction, a similar fraction of differential peaks falls in regions of CNAs for a single cell line. However, two observations suggest that the DiffBind results may be more accurate. First, we compare NRF1 binding in K562 cells vs. H1hesc, the former having 16.2% of peaks in CNA regions and the latter only 0.3%. Differential peaks identified either with or without input correction are enriched in K562 CNA regions (25 vs. 16.2%). However, we see higher enrichment for differential binding in CNA regions when considering sites only identified without input correction (30 vs. 25%). This suggests that correcting for input identifies fewer differential-binding sites in CNA regions. Second, a comparison of HEK293 vs. MCF7 finds a similar result. Here, both cell lines have copy number alterations (36 and 28% of genome, respectively) so the comparison is indirect. With or without input correction, around 33% of the differential peaks are found in regions of CNA for HEK293 and around 27% of the differential peaks in regions of CNA for MCF7 (Supplemental Table 5). However, focusing once more on differential peaks only found without input correction we see a higher than expected number occurring in CNA regions for HEK293 (44 vs. 33%), and a lower than expected number in CNA regions for MCF7 (20 vs. 27%, or more normal copy regions). Together, these indicate the regions identified when input is not corrected are enriched in sites with CNAs between HEK293 and MCF7. We conclude from this that subtracting input using DiffBind can potentially improve accuracy of detecting differential binding in regions where copy number is different.

#### MAnorm3

**Figure 5:**
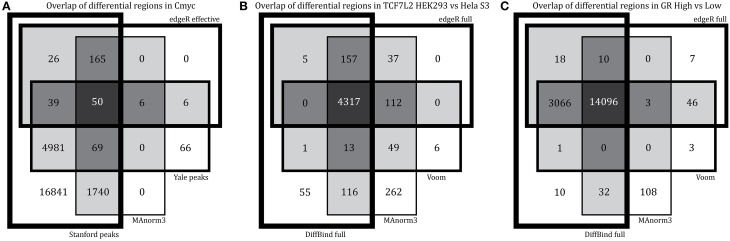
**Differences in results of sample-level comparison methods**. We overlap the regions with significant differential binding found by each method for **(A)** c-Myc, **(B)** TCF7L2 HEK293 vs. Hela S3, and **(C)** GR high vs. low hormone. For c-Myc (our negative control), we picked methods with highest number of false-positives and overlap these results with the peaks found by peak caller (Yale and Stanford).

This method applies edgeR after normalizing using peaks that are shared between conditions. When applied to the control datasets, this method finds no differences in Pol2 but finds a surprisingly high number of differentially bound regions in c-Myc (9% of total) (Table 3). This result remained unchanged after down-sampling c-Myc samples to similar sequencing depth and/or only using the top 5000 peaks from each dataset (data not shown). Most differential regions identified by MAnorm3 in c-Myc are unique to the Stanford data set with higher sequencing depth (Figure 5A). Although we originally selected c-Myc as a negative control, this small fraction of differentially bound regions suggests either that MAnorm3 did not properly account for differential sequencing depth when total binding is unchanged, or that some of the peaks with highest occupancy changed during the lag time between experiments, an explanation also considered earlier from our overlap analysis. We used our *in-silico* derived Pol2 datasets of 8 M and 46.2 M reads once more to assess the effect of different sequencing depth on the false-positive rate for experiments run at the same time. After normalizing on shared peaks we found less than 1% false-positives, showing that MAnorm3 can correct for differential sequencing depth when total binding is the same between conditions.

When comparing between cell types, the MAnorm3 strategy performs comparably with the other sample-level comparison methods but has the highest number of unique differential regions (TCF7L2 results shown in Figures 4F, 5B). The GR dataset violates MAnorm3's assumption that most shared peaks are not differentially bound since more binding is expected with higher hormone treatment. Thus, normalizing using MAnorm3 removes true binding differences in shared peak regions and has reduced sensitivity for GR dataset (Figure 5C) with over 3000 differential binding regions identified by all other methods missed by MAnorm3. We believe that MAnorm3 can be useful in circumstances when average binding for peaks shared between samples is unchanged, but caution that the results are very sensitive to this assumption and recommend full library size normalization when the assumption is not met.

#### Voom

This method produces similar or slightly fewer significant differential regions compared to edgeR with full library size (Table 3, Supplemental Table 3). MA plots show that this Voom transformation greatly reduces the read counts (Figure 4I). Overall, the similar results obtained for these data sets suggest Voom may be a useful alternative approach for differential TF binding analysis that would open access to methodology developed for gene expression microarrays, for example gene set testing.

### Comparing reproducibility of differential binding results across analysis approaches

**Figure 6:**
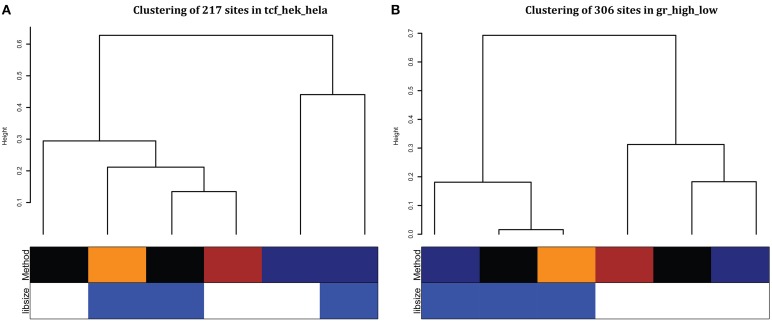
**Clustering of fold changes from top differentially bound regions from each method**. We combine the top 100 differential peaks in each condition and cluster estimated fold changes. We used hierarchical clustering with average linkage and Gower's distance on log2-fold changes weighted based on how often that region was significant. **(A)** TCF7L2 HEK293 cell type vs. HeLa S3 cell type, **(B)** GR high vs. low comparison. For the Method row: edgeR (black), DiffBind (dark blue), MAnorm3 (brown), and voom full (orange). For the libsize row: effective library size (white) and full library size (blue).

To evaluate the differences between the sample-level comparison methods, we cluster the fold change estimates for the top differentially bound sites for different methods with more weight on the regions that were more robustly differentially regulated (Table 3). For cell-type comparisons, TCF7L2 showed that methods clustered based on whether input was subtracted (Figure 6A). Since two of the three TCF7L2 cell types were cancer cell lines, input subtraction can help remove effects from copy number differences due to chromosomal abnormalities in these cancer samples. All but one of the NRF1 fold changes showed input subtraction and Voom clustering together (Supplemental Figure 2). The comparison that did not cluster based on input subtraction was between two non-cancer cell lines (GM12878 and H1 ESC) further suggesting the importance of input subtraction for cancer cell lines.

For hormone treatment comparisons, normalization makes the biggest difference on fold change estimates (GR in Figure 6B) and samples using full library size (blue) cluster together when comparing experiments with a large differential in numbers of peaks found. One comparison that did not cluster by normalization method found a large overlap in number of differential regions (ERα est vs. gen) (Supplemental Table 3 and Supplemental Figure 2).

### Validation of fold changes

We validated fold changes by performing ChIP-qPCR on GR peaks and using the validation data from the supplement of the published TCF7L2 analysis (Frietze et al., 2012) (also S. Frietze, personal correspondence) (Supplemental Figure 3). As expected for the GR dataset, using full library size parameter performs closest to qPCR validation when comparing between high vs. low hormone treatment with edgeR performing better than DiffBind. For TCF7L2 datasets, the fold change estimates from all different methods are comparable to qPCR data at validated regions. We conclude that of the methods we considered, edgeR with full library size estimates fold changes closest to qPCR validation under most conditions, but the variation in results suggests further improvements on normalization methods is warranted.

## Discussion

A number of new software packages for performing differential TF binding analysis are publicly available, without accompanying papers that assess their performance. These approaches build on mature RNA-seq methods (Rapaport et al., 2013) to perform differential binding analysis on known protein binding regions. Although a larger number of methods allow the comparison of pairs of experiments (Zhang et al., 2008; Heinz et al., 2010; Huang et al., 2011; Taslim et al., 2011; Bardet et al., 2012), those reviewed here analyze biological replicates (Stark and Brown, 2011; Liang and Keles, 2012) since accurate identification of real differential binding is likely to rely more on the number of biological replicates than sequencing depth (Rapaport et al., 2013). In addition, to specifically compare statistical modeling approaches we standardize a number of analysis steps, such as peak calling and read counting over features.

We evaluate methods that correct for non-specific binding and use different normalization methods to account for sequencing depth differences between samples. This differs from normalization performed during ChIP-seq peak calling to account for features such as input and GC content, whereas identifying differential peaks requires additional normalization to make the different ChIP-seq experiments comparable. The lack of true gold-standard data sets makes a general comparison of methods difficult. Nevertheless, the sensitivity of results to different methods under different biological comparisons highlights proper normalization as a key analysis step and identifies issues that bioinformaticians need to know about the data, such as the expected differences in DNA-protein binding between conditions and sample chromosome copy number.

Of the analysis steps evaluated, normalization for sequencing depth between samples had the greatest potential for influencing differential binding site discovery. ChIP-seq peak callers can also be sensitive to read depth, with more reads leading to more peaks found even when using the same FDR and IDR cutoff, as seen in the c-Myc and the *in silico*-created Pol2 control datasets. Normalization for sequencing depth is a crucial step that could lead to different conclusions if ignored or performed incorrectly. Large differences in DNA-protein binding between conditions causes an imbalance in number of peaks found and will skew the signal to noise ratio between conditions. Knowing whether biological differences in the amount of DNA bound protein targeted by the ChIP antibody are expected between conditions is crucial for choosing the best normalization method. For example, in the GR hormone treatment dataset, using full library size (all reads) for normalization produced results that were consistent with the expectation of higher binding with higher hormone concentration and confirmed in our ChIP-qPCR validation.

Given that sample input is often critical for peak calling, background correction (specifically, input subtraction) had a modest influence on the identification of differential binding sites. However, we note that the impact could be greater when comparing samples with more extreme variation in copy number. Using DiffBind, we found some differences in regions with high input leading to changes in ranks of differential binding sites, their fold-change estimates, and the number of differential binding sites discovered for comparisons within the same cell-type. We found little differences in the number of significant differential binding sites identified in the comparisons between cell types, however differences that were observed occurred more often in regions with CNAs. Future work could investigate how these results compare to alternate CNA adjustment methods such as ABCD-DNA (Robinson et al., 2012).

A major difference between ChIP-seq and RNA-seq is that counts for differential RNA-seq are obtained over features (genes) defined independently of the mapped sequence reads. These features are shared between all treatment conditions, whereas counts for differential ChIP-seq are obtained over peaks defined from the sequencing reads. In this paper we analyzed differential binding from merging peak found in our pair-wise comparisons, a common practice for current differential binding analyses (Stark and Brown, 2011; Liang and Keles, 2012; Shao et al., 2012). Recent work suggests that FDR control can be lost when merging peaks between conditions and instead proposes peak calling for binding region discovery from the set of reads pooled across experimental conditions (Lun and Smyth, 2014). Although this different approach may affect individual results from our analyses, we do not believe it would change our conclusions about the steps in the data processing pipeline having the greatest impact on differential binding results.

Differential peak calling methods are useful when comparing binding both between different cell types and between the same cell types after exposure to different treatments. As more ChIP-seq datasets are published with multiple conditions, interest in quantifying binding between conditions will only be increased. Our study provides an overview of the differential ChIP-seq binding analysis workflow and illustrates for experiments with different amounts of total protein bound the potential poor performance of data normalization using methods that do not consider the full number of reads sequenced. We recommend performing differential binding on datasets with similar sequencing depth; using edgeR with full library size normalization when total binding differs between experiments; and subtracting input using DiffBind when comparing between cells with extensive known aneuploidy.

## Author contributions

All authors read and approved of the manuscript for publication. DW conceived of the study and drafted the manuscript with assistance from KS and MS. KS assisted with data analysis and study design. MS and DB advised on biological interpretation and assisted in proofreading the manuscript. DB performed ChIP-qPCR validation.

### Conflict of interest statement

The authors declare that the research was conducted in the absence of any commercial or financial relationships that could be construed as a potential conflict of interest.

## Abbreviations

ChIP: chromatin immuneprecipitation
qPCR: quantitative PCR
GR: glucocorticoid receptor; ERα, Estrogen Receptor alpha
CNAs: copy number alterations
NCIS: Normalization of ChIP-seq
CPM: counts per million
TMM: trimmed mean of M values.
