# Supplementary material for: Identifying differential transcription factor binding in ChIP-seq
Source: Front Genet. 2015 Apr 29;6:169. doi: 10.3389/fgene.2015.00169 (PMC4413818; doi:10.3389/fgene.2015.00169)
Supplement: Supplementary file 1 [file Presentation1.PDF]

Supplemental Figure 1 Overlapping peaks from different conditions

NRF1 Overlaps

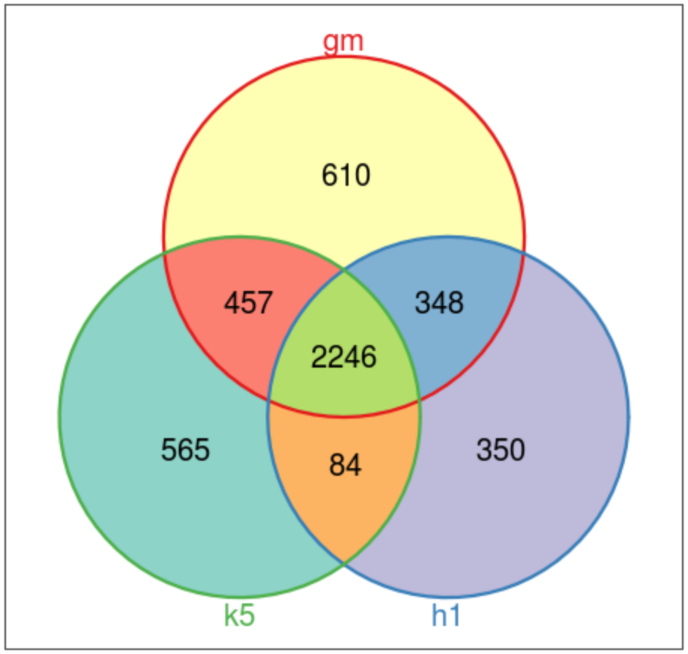

ERα Overlaps

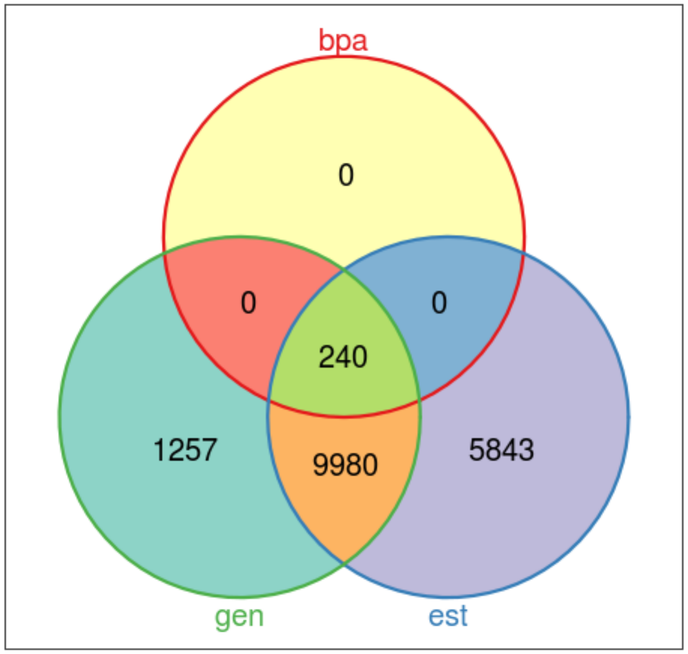

Caption: This figure shows peaks from different conditions that overlap more than one base pair. NRF1 overlaps (left) shows peaks from Gm12878, H1 hESC, and K562 cell lines. ERα Overlaps (right) show peaks from BPA, estradiol, and genistein.

Supplemental Figure 2 Clustering of top peaks in each pairwise comparison

We take the top 100 peaks found by each differential peak calling method and generate a list of unique peaks after combining the top peaks from each method. We then cluster over the fold changes for this list of unique peaks putting more weight on peaks that were found by many methods

Legend:

Method:

edgeR : black
DiffBind: purple
MAnorm: brown
voom : orange

libsize:

effective library size : white
full library size: blue

Cell-type

TCF7L2

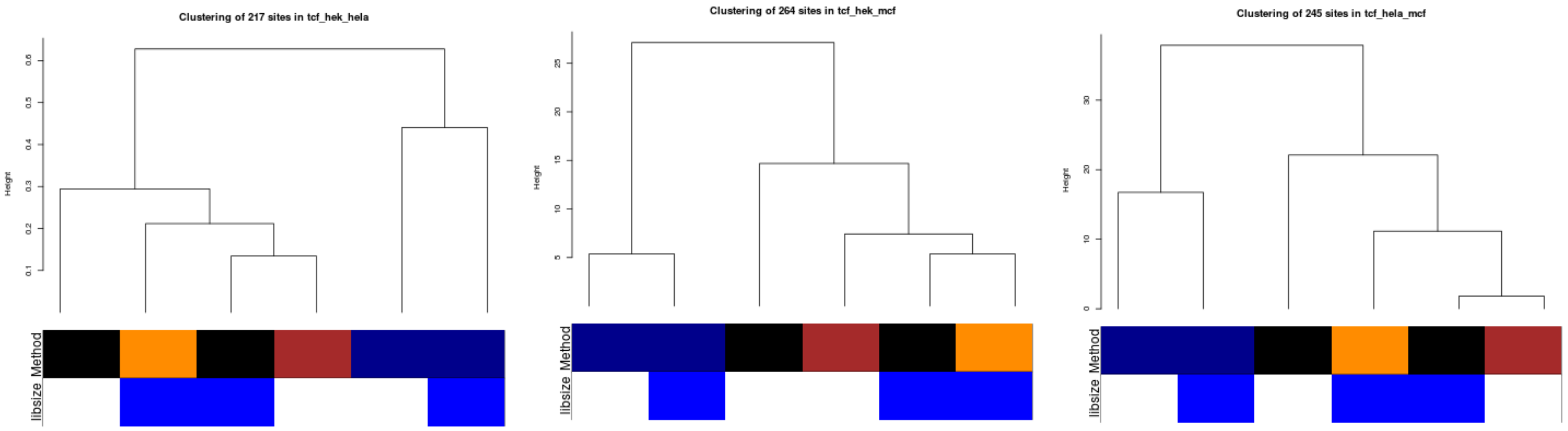

Caption: When comparing TCF7L2 binding between different cell types, top peaks cluster most distinctly based on if input is or is not subtracted (DiffBind, purple box), highlighting the importance of input subtraction between these different cell types.

NRF1

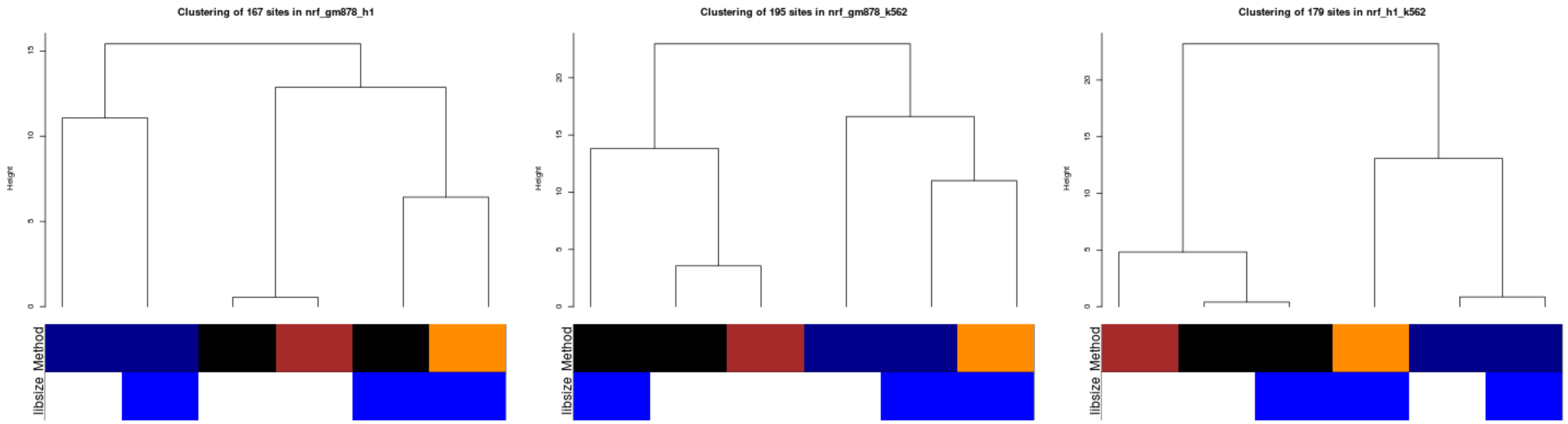

Caption: When comparing NRF1 binding between different cell types, top peaks from MAnorm and edgeR using effective library size are most similar to each other with input subtraction (DiffBind, purple box) and voom (orange box) clustering separately.

Treatment

GR

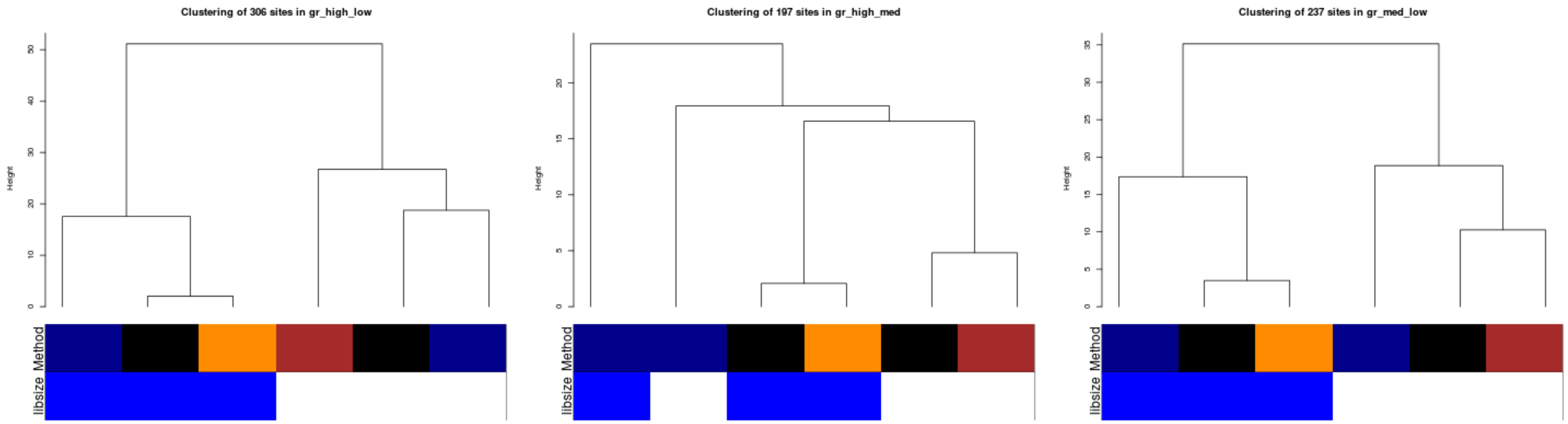

Caption: When comparing GR binding between different hormone concentrations, top peaks are clustered based on full library normalization (blue) versus effective library normalization (white) for comparisons between treatment dosages with large differences in total binding (high or mid dose compared to low dose). The impact of library size normalization was reduced for the only comparison where GR binding is expected in both conditions (high vs mid dose). This highlights the importance of choosing the correct normalization procedure for differential binding analysis.

ERα

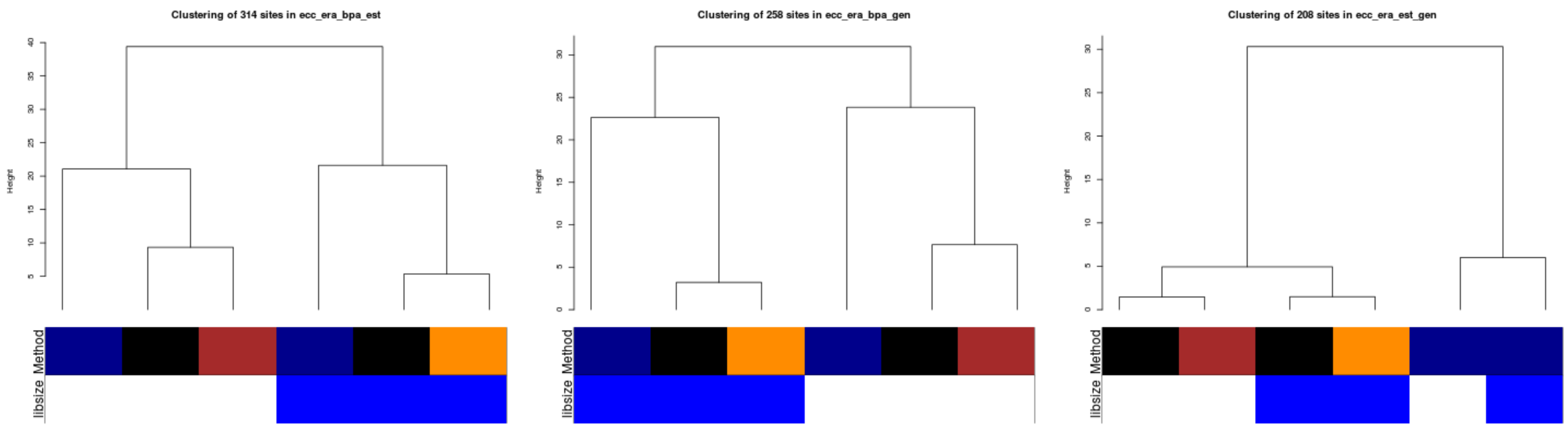

Caption: When comparing ERα binding between different hormone treatments, top peaks are clustered based on full library normalization (blue) versus effective library normalization (white) with the exception of est vs gen where input subtraction (Diffbind, purple box) forms an outgroup. This could suggest genistein inducing similar ER binding patterns to estradiol.

Figure 3 PCR validation of GR and TCF7L2 datasets

Note that for the data presented, the GR ChIP-qPCR was performed on samples using the same cell type and antibody and following the published protocol as closely as possible, but the qPCR experiment used different DNA than the ChIP-seq and different lot number for antibody, which could introduce additional variability. In contrast, the TCF7L2 ChIP-qPCR was performed on the same DNA as ChIP-seq so we expect and find fold change estimates to match more closely.

Fold changes from ChIP-qPCR are compared to fold changes calculated from each of the sample-level comparison methods listed above with the addition of a new input subtraction method called Normalization of ChIP-Seq (NCIS) that scales input based on non-enriched regions [1]. This method is implemented by the DBChIP package (version 1.2.0) [2] and was used to evaluate differences in input scaling. Unlike edgeR and DiffBind that allow the user to specify the binding regions and library size, DBChIP clusters input peaks and uses a median ratio for library size. Using the median ratio with and without background subtraction after NCIS scaling is visualized as NCIS sub and NCIS median.

Legend:

Fold change different boxplot horizontal axis:

edgeR effective library size

edgeR full library size

DiffBind effective library size

DiffBind full library size

DBChIP using median normalization

DBChIP using median normalization + subtract input

MAnorm3

Voom using full library size

Scatterplot symbols key

ChIP-qPCR – solid black circle

edgeR with effective library size – black square

edgeR with full library size – blue square

DiffBind (subtract input) with effective library size – black triangle

DiffBind (subtract input) with full library size – blue triangle

DBChIP (NCIS) with median library size – black diamond

DBChIP (NCIS) with median library size and subtract scaled input – green diamond

MAnorm + edgeR (MAnorm3) – black X

Voom using full library size – blue X

Barplot symbols

Asterisks (*) next to site number indicate significant fold change difference (FDR adjusted p-value < 0.05) for that site using edgeR w/full library size and merge pairwise reference binding regions

<u>GR</u>

SITE	CHR	START	END	GENE	REASON	PCR primer F	PCR primer R
1	chr17	8125597	8125653	LINC00324	mid only, low enrichment, some input	gcggggatactcaccactatac	TTCGGAGAAAGGTCTCTACAGG
2	chr8	96113346	96113480	-	mid only, lots of high binding (no peak)	TGTGTTCTTAACAGGGCAAAGA	GCAACATTGTGCATTATGTGACT
3	chr8	96113623	96113736	-	high only, some mid binding (no peak)	tccacaacatacctgagagtgg	cttccagcaattcagtgttcaa
4	chr16	2136211	2136292	WDR45B	high only, some mid binding	CATCCTGTCCAATGAGCATGG	GCAGTCCTTCAGCTCGATGA
5	chr5	43067098	43067170	LOC100506639	high + low peak, low enrichment	TTTACCTATTGGTGCTCGTGTG	GCCGAAGTGTACTAGGTTCCAG
6	chr17	57903034	57903128	VMP1	high + med peak, induced	AATCATTGAACTTGAGGGCTGG	TAGGAGACAGCAAAGAAGAGCA
7	chr8	48266351	48266460	SPIDR	common peak	ATTCAGTGACGAAGATTGCTGG	ACATGCAAAGACAAGAGGAACA
8	chr12	6485519	6485662	SCNN1A	common peak, induced	caccttcagtgcctgctttc	aggccaggaatgtgtaatcg
9	chr10	74008697	74008772	-	common peak, induced	CCATGTGCAGTTCTCTTCTTCA	CCGGATAAGTTCTCAGCCAGA
10	chr5	159247191	159247275	-	common peak, induced	CCTTGCTTTCTCACACTTTCCA	GCTTCCCTGATATTTCCACACC

High vs low

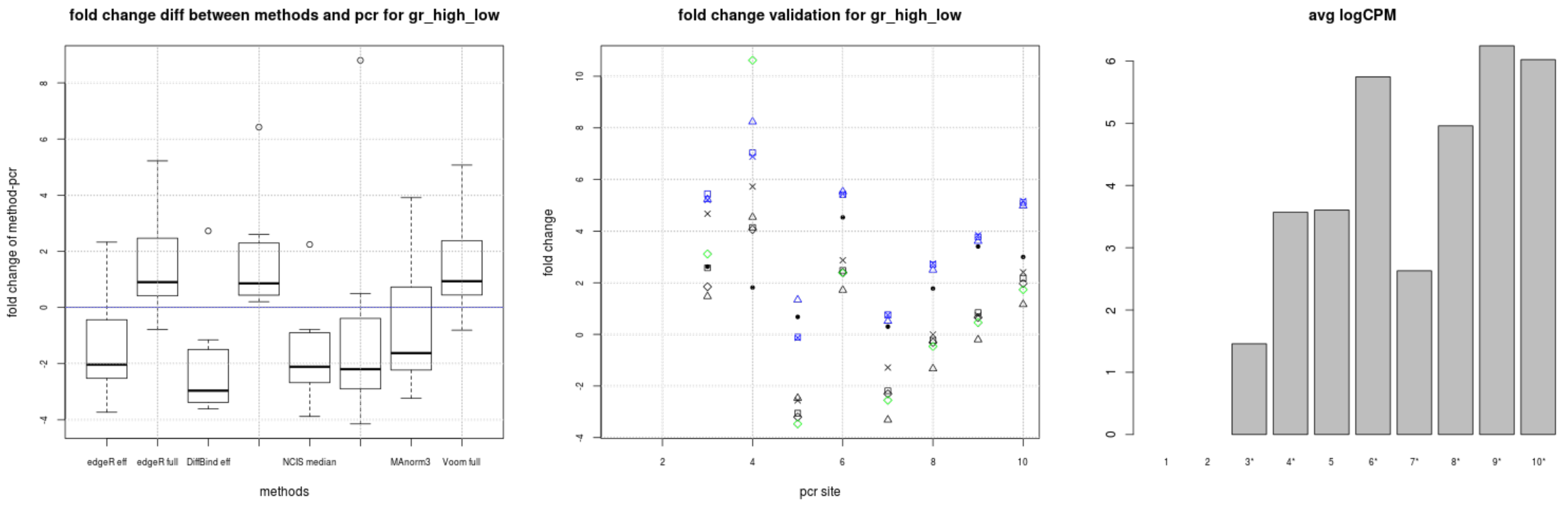

Caption: In this validation of GR binding between high vs low, we find that methods using full library size performs closest to qPCR validation with methods using effectively library size consistently underestimating the fold change.

High vs med

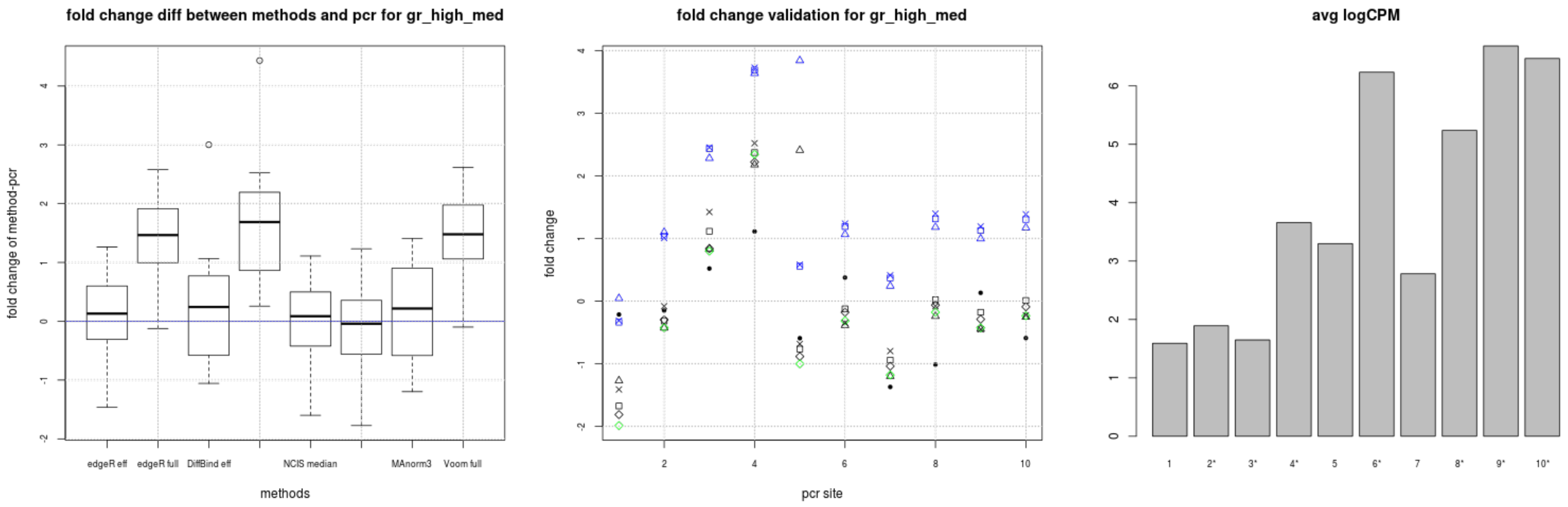

Caption: In this validation of GR binding between high vs med, we are surprised to find that effective library size performs better. This could be due to higher levels of GR hormone treatment performing well with effective library size. Unfortunately, most of the qPCR validation results were less than 2 fold change and it is possible some methods may have performed better at different peaks with larger fold changes.

Med vs low

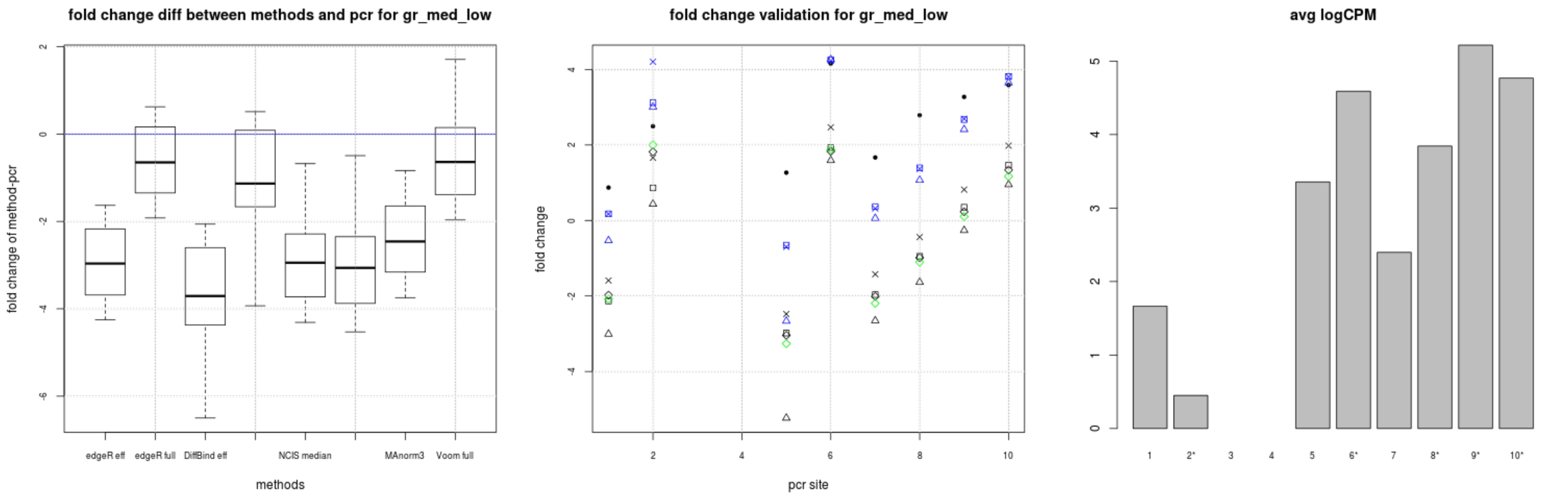

Caption: In this validation of GR binding between med vs low, we find that methods using full library size perform better than effective library size methods. Nevertheless, we find that most fold changes are underestimated – especially sites 5-10 with more binding (higher logCPM).

TCF7L2

SITE	CHR	START	END	REGION	REASON	PCR primer F	PCR primer R
1	chr19	44980743	44980879	ZNF180 3'exon	Negative	TGATGCACAATAAGTCGAGCA	TGCAGTCAATGTGGGAAGTC
2	chr19	6577759	6577870	CD70 downstream	Negative	TCTGCCAGTGGAAGTGTTTG	GCTGGGGTCTTCTGGTCTTA
3	chr5	24647111	24647222	CDH10 proximal	Negative	AGAAAGTTGCTCCCGTCCTT	GCCTCCCTAGCAACAATGTG
4	chr12	71834603	71834713	LGR5 proximal	Common	TTTTCCTCCCTTCCTTCCTC	CTAATCGGGGTTTGTGGTTG
5	chr20	52824448	52824554	PFDN4 proximal	Common	TCCTTCCAGGTCACATTTCC	GAACTCCCCGCAAAAATACA
6	chr20	26189969	26190118	miR663 proximal	Common	GCGTCTCGTCTCACTCAACC	GGGTTGTCCCTCAGGTTCC
7	chr1	113007079	113007209	WNT2B distal	MCF7	GCCTGGCCTGTACAGAAAGT	CAGGTGAGAGGGAAGGAGTG
8	chr12	121665236	121665377	P2RX4 intron	MCF7	GGTGCTTTGTGCACTTTTCA	AATCAGTAGGCCTGGCTTTG
9	chr20	53265505	53265648	DOK5 intron	MCF7	TGCCTTGGAAGATAGCATCA	CTACATGGAAGACGCCTGGT
10	chr5	125721829	125721931	GRAMD3 intron	HeLaS3	TTTGGTTGACGTAGATGCTGA	TGATCCGTGATGTCCAGCTA
11	chr5	32028199	32028307	PDZD2 intron	HeLaS3	GGTGCAACCAGTCCTGAAGT	GCTCTTGCAGCCTTACGAAC
12	chr5	39759879	39760018	DAB2 distal	HeLaS3	GGTTTGCTGAGCTGAGTTTTG	TTTGATCTGACAATTCCCTTTTC
13	chr12	83255933	83256023	TMTC2 intron	HEK293	GGGGAAAAGCCCTTGAAATA	GGGGTCATGAGAGGCTTGTA
14	chr20	7066009	7066134	PLCB1 distal	HEK293	GGTGGCTGCCTTATCTTTCA	TTAAAAGGCATCCCACCAAG
15	chr19	19541726	19541859	GATAD2A intron	HEK293	CAAGGCTGATCTTCCCTCAG	ATGGTTTGGCTTTGACCTTG

The PCR validation data for these sites were taken from Frietze et al’s 2012 Genome Biology paper (in supplemental data and also personal correspondence).

HEK293 vs HeLa S3

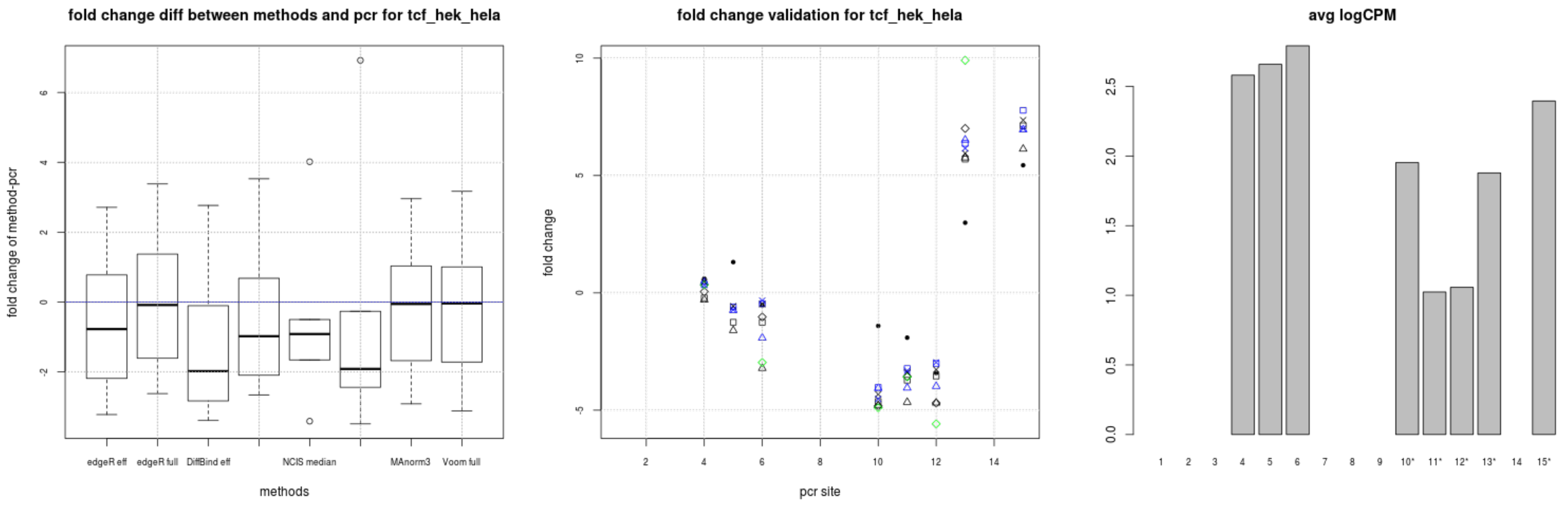

Caption: In this validation of TCF7L2 binding between Hek293 and Hela S3 cell types, we find that most methods perform similarly although fold changes tend to be overestimated for the truly differential sites (10-13)

HEK293 vs MCF7

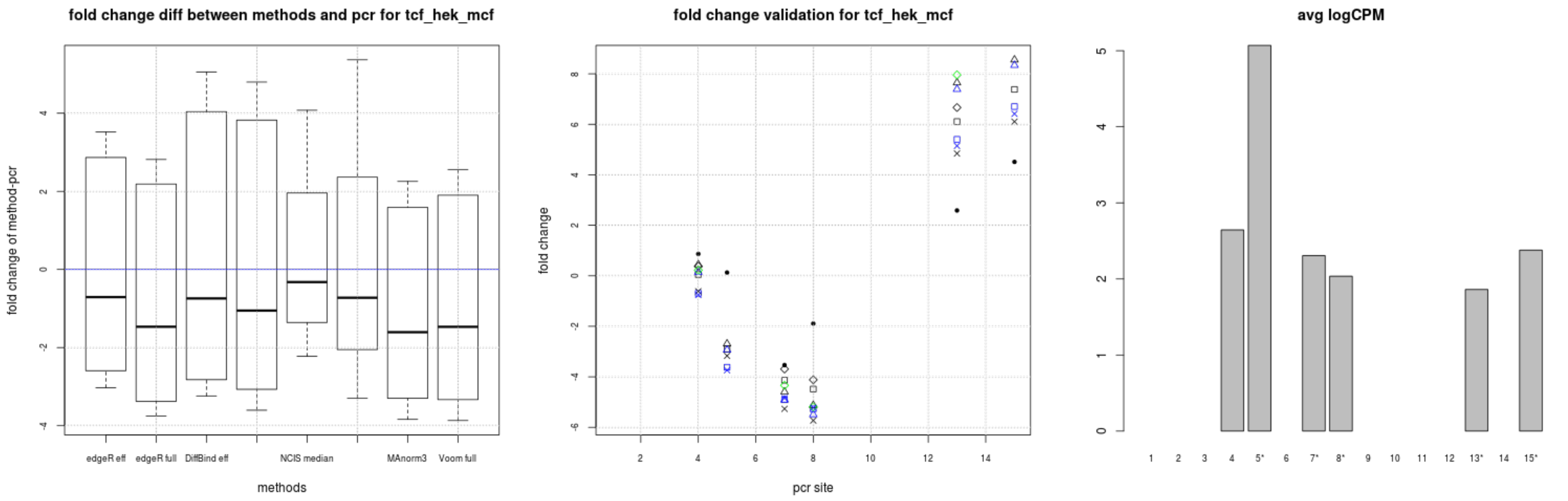

Caption: In this validation of TCF7L2 binding between Hek293 and Mcf7 cell types, we find that most methods perform similarly poorly with fold changes once again overestimated in most of the truly differential sites (7,8,13,15)

HeLa S3 vs MCF7

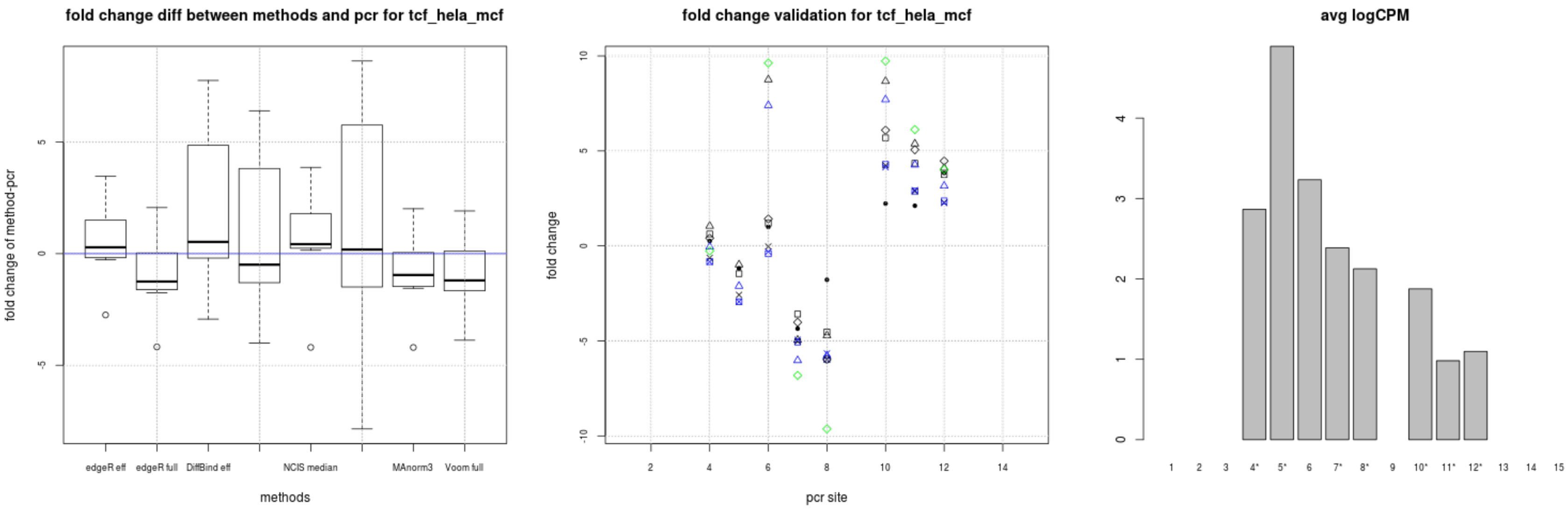

Caption: In this validation of TCF7L2 binding between Hela S3 and Mcf7 cell types, we find that edgeR with effective library size and DBChIP performed well with DBChIP with input subtraction performing especially poorly. The poor results from the boxplots are mostly due to sites 6 and 10 where input subtraction methods (DiffBind and DBChIP with subtraction) largely overestimate the fold changes.

Supplemental Table 1

Availability of data

Peaks: http://hgdownload.cse.ucsc.edu/goldenPath/hg19/encodeDCC/wgEncodeAwgTfbsUniform/

Reads: http://ftp.ebi.ac.uk/pub/databases/ensembl/encode/users/anshul/humanENCODE/rawdata/mapped/mar2012/tagAlign/

Reads can also be generated from bam files using bamtobed

Signal: http://www.broadinstitute.org/~anshul/projects/encode/rawdata/signal/mar2012/pooledReps/bigwig/macs2signal/logLR/

Details about IDR can be found here: https://sites.google.com/site/anshulkundaje/projects/idr

blacklist: http://hgdownload.cse.ucsc.edu/goldenPath/hg19/encodeDCC/wgEncodeMapability/wgEncodeDacMapabilityConsensusExcludable.bed.gz

Details about blacklist: https://sites.google.com/site/anshulkundaje/projects/blacklists

CNV: http://hgdownload-test.cse.ucsc.edu/goldenPath/hg19/encodeDCC/wgEncodeHaibGenotype/

The file name prefixes for the files as well as shift length and number of peaks found can be found below. All reads were generated by Illumina single end sequencing without duplicates removed. Duplicate removal resulted in a limitation in the maximum number of reads within a peak.

shorthand	ENCODE dataset	Read length	shift length	filtered reads	# peaks
**ctrl**					
cmyc_s	wgEncodeSydhTfbsK562CmycIggrabAlnRep1	27	55	17454841	24153
	wgEncodeSydhTfbsK562CmycIggrabAlnRep2	32	58	22170003	
cmyc_y	wgEncodeSydhTfbsK562CmycStdAlnRep1	27	43	4074225	5023
	wgEncodeSydhTfbsK562CmycStdAlnRep2	27	50	3952967	
pol2_odd	wgEncodeSydhTfbsGm12891Pol2IggmusAlnRep1	34	68	12155544	15863
	wgEncodeSydhTfbsGm12891Pol2IggmusAlnRep3	34	68	14002156	
	wgEncodeSydhTfbsGm12891Pol2IggmusAlnRep5	28	70	7235580	
pol2_even	wgEncodeSydhTfbsGm12891Pol2IggmusAlnRep2	34	68	13634348	17362
	wgEncodeSydhTfbsGm12891Pol2IggmusAlnRep4	28	78	6431115	
	wgEncodeSydhTfbsGm12891Pol2IggmusAlnRep6	28	70	7119824	
pol2_pool					20390

**treat**					
gr_low	wgEncodeHaibTfbsA549GrPcr1xDex500pmAlnRep1	36	55	19631993	100
	wgEncodeHaibTfbsA549GrPcr1xDex500pmAlnRep2	36	53	15082857	
gr_high	wgEncodeHaibTfbsA549GrPcr1xDex50nmAlnRep1	36	60	19268264	17508
	wgEncodeHaibTfbsA549GrPcr1xDex50nmAlnRep2	36	70	16744013	
gr_med	wgEncodeHaibTfbsA549GrPcr1xDex5nmAlnRep1	36	60	20102893	4540
	wgEncodeHaibTfbsA549GrPcr1xDex5nmAlnRep2	36	63	20540890	
era_bpa	wgEncodeHaibTfbsEcc1EraaV0416102Bpa1hAlnRep1	36	58	13191238	238
	wgEncodeHaibTfbsEcc1EraaV0416102Bpa1hAlnRep2	36	53	19623381	
era_est	wgEncodeHaibTfbsEcc1EralphaaV0416102Est10nm1hAlnRep1	36	65	11852078	16091
	wgEncodeHaibTfbsEcc1EralphaaV0416102Est10nm1hAlnRep2	36	68	11185788	
era_gen	wgEncodeHaibTfbsEcc1EralphaaV0416102Gen1hAlnRep1	36	60	9195577	11452
	wgEncodeHaibTfbsEcc1EralphaaV0416102Gen1hAlnRep2	36	63	11062229	
**Type**					
tcf_hek	wgEncodeSydhTfbsHek293Tcf7l2UcdAlnRep1	32	103	22281421	4003
	wgEncodeSydhTfbsHek293Tcf7l2UcdAlnRep2	32	95	22118023	
tcf_hela	wgEncodeSydhTfbsHelas3Tcf7l2UcdAlnRep1	32	145	14693303	3198
	wgEncodeSydhTfbsHelas3Tcf7l2UcdAlnRep2	32	125	10101457	
tcf_mcf	wgEncodeSydhTfbsMcf7Tcf7l2UcdAlnRep1	32	100	20415125	6550
	wgEncodeSydhTfbsMcf7Tcf7l2UcdAlnRep2	32	115	22652659	
nrf_gm	wgEncodeSydhTfbsGm12878Nrf1IggmusAlnRep1	34	40	12231442	3660
	wgEncodeSydhTfbsGm12878Nrf1IggmusAlnRep2	27	50	25653087	
nrf_h1	wgEncodeSydhTfbsH1hescNrf1IggrabAlnRep1	27	55	20534959	3027
	wgEncodeSydhTfbsH1hescNrf1IggrabAlnRep2	27	50	23858547	
nrf_k5	wgEncodeSydhTfbsK562Nrf1IggrabAlnRep1	34	48	14404504	3353

wgEncodeSydhTfbsK562Nrf1IggrabAlnRep2	34	68	13133792

The input files associated with the conditions listed above can be found below. These input files are used for input subtraction methods.

associated samples	input	filtered reads
pol2_odd, pol2_even	wgEncodeSydhTfbsGm12891InputIggmusAlnRep0	14213452
cmyc_s, cmyc_y	wgEncodeSydhTfbsK562InputStdAlnRep0	31081386
gr_high, gr_med, gr_low	wgEncodeHaibTfbsA549GrPcr1xEtoh02AlnRep0	40725818
era_bpa, era_est, era_gen	wgEncodeHaibTfbsEcc1EralphaaV0416102Dm002p1hAlnRep0	23396020
tcf_hek	wgEncodeSydhTfbsHek293InputUcdAln	35647336
tcf_hela	wgEncodeSydhTfbsHelas3InputUcdAln	26714432
tcf_mcf	type/wgEncodeSydhTfbsMcf7InputUcdAlnRep0.bam.unique.tagAlign.gz	26527086
nrf_gm	wgEncodeSydhTfbsGm12878InputIggmusAlnRep0	8822988
nrf_h1	wgEncodeSydhTfbsH1hescInputIggrabAln	35304723
nrf_k5	wgEncodeSydhTfbsMcf7InputUcdAlnRep0	27953841

Supplemental Table 2

MAnorm3 can be found here: https://github.com/ying-w/chipseq-compare/tree/master/Manorm

Changes include:

- Uses edgeR for differential analysis and can have 2x replicates per comparison
- Estimate MA from more than two samples
- Runs bedtools in parallel to greatly speed up computational time
- Creates a folder for each analysis

Supplemental Table 3

**Treatment**	GR High vs Low	%	GR High vs Med	%	GR Med vs Low	%	ERα bpa vs est	%	ERα bpa vs gen	%	ERα est vs gen	%
Non-Overlap	17339	99	12960	74	4441	98	15730	99	11164	98	7075	41
edgeR efflib	4318	25	387	2	912	20	223	1	0	0	335	2
edgeR fulllib	17246	99	11305	65	4289	95	10986	69	3048	27	2	0
DiffBind efflib	2908	17	396	2	819	18	9	0	0	0	429	2
DiffBind fulllib	17233	99	8304	48	3939	87	9063	57	663	6	110	1
MAnorm3	14249	82	1535	9	1645	36	897	6	0	0	320	2
voom fulllib	17215	99	11196	64	4111	91	10914	68	2467	22	3	0
# peaks	17439		17459		4536		15968		11403		17202	

Caption: This table of differential peaks illustrates the difference between full library size and effective library size normalization in treatment effects. With the exception of ERα est vs gen, full library size had more differential binding sites than effective library size. For ERα, non-overlap finds more differential regions than methods using replicates suggesting the differential peaks found by overlapping might not be robust.

**Cell-Type**	TCF Hek vs Hela	%	TCF Hek vs Mcf	%	TCF Hela vs Mcf	%	NRF1 Gm vs H1	%	NRF1 Gm vs K562	%	NRF1 H1 vs K562	%
Non-Overlap	5314	89	8429	91	6744	85	1497	37	1601	37	1717	42
edgeR efflib	5199	87	7745	84	5770	73	1687	41	2114	49	2382	59
edgeR fulllib	4627	77	7944	86	6674	84	1738	43	2011	47	1098	27
DiffBind efflib	5238	88	7744	84	5859	74	1732	42	2106	49	2295	57
DiffBind fulllib	4663	78	7547	82	6532	82	1594	39	1722	40	1086	27
MAnorm3	5063	85	8411	91	6869	87	1638	40	2021	47	2363	58
voom fulllib	4496	70	7803	70	6599	70	1206	29	1456	34	791	20
# peaks	5976		9260		7931		4089		4297		4041	

Caption: The above table illustrates the similar number of differential regions found between different methods for comparisons between cell types. Subtracting input sometimes finds less regions and using Voom method will always find the least number of differential regions.

Controls	Pol2 Odd vs Even	%	Cmyc Stanford vs Yale	%
Non-Overlap	4885	30	17962	79
edgeR efflib	0	0	292	1
edgeR fulllib	0	0	0	0
DiffBind efflib	5	0	411	2
DiffBind fulllib	46	0	7	0
MAnorm3	0	0	1991	9
voom fulllib	0	0	1	0
# peaks	16278		22828	

Caption: The above table illustrates the number of differential regions found using various methods for our negative control datasets. We would expect very few sites to be differential and the only large deviation from this was using MAnorm on the Cmyc sample where 9% of the peaks were found to be differential.

Supplemental Table 4: Copy number variation within peaks

TF	Cell Type	% of array is cnv (in base pairs)	No. of peaks with CN calls	% of peaks in cnv	Cell Type
TCF7L2	Hek293	35.5%	3274	34.0%	embryonic kidney
TCF7L2	HelaS3	25.3%	2851	16.7%	cervical carcinoma
TCF7L2	MCF-7	28.0%	5909	24.9%	mammary gland, adenocarcinoma
NRF1	Gm12878	8.6%	2715	5.0%	immortalized blood cell
NRF1	H1hesc	0.1%	2321	0.3%	embryonic stem cells
NRF1	K562	27.3%	3054	16.2%	leukemia

This table shows copy number variation data from Illumina Human 1M-Duo Infinium HD BeadChip assay and circular binary segmentation (CBS) and the number and percentage of peaks in regions with non-normal copy number (provided by ENCODE).

Supplemental Table 5

	edgeR full # diff peak	edgeR full # with CNV calls	edgeR full # in CNV region	edgeR full % sig in CNV	DiffBind full # diff peak	DiffBind full # with CNV calls	DiffBind full # in CNV region	DiffBind full % sig in CNV	Significant in edgeR not DiffBind # diff peak	Significant in edgeR not DiffBind # with CNV calls	Significant in edgeR not DiffBind # in CNV region	Significant in edgeR not DiffBind % sig in CNV
tcf hek vs hela (hek)	4627	3797	1316	0.347	4663	3832	1329	0.347	184	155	54	0.348
tcf hek vs hela (hela)	4627	4078	945	0.232	4663	4119	947	0.230	184	167	31	0.186
tcf hek vs mcf (hek)	7944	6612	2185	0.330	7547	6301	2067	0.328	659	326	143	0.439
tcf hek vs mcf (mcf)	7944	7111	1917	0.270	7547	6747	1812	0.269	659	538	109	0.203
tcf hela vs mcf (hela)	6674	5958	1298	0.218	6532	5814	1249	0.215	635	481	156	0.324
tcf hela vs mcf (mcf)	6674	6058	1587	0.262	6532	5924	1552	0.262	635	488	107	0.219
nrf gm vs h1 (gm)	1738	1282	61	0.048	1594	1168	57	0.049	201	137	7	0.051
nrf gm vs h1 (h1)	1738	1354	2	0.001	1594	1253	1	0.001	201	148	0	0.000
nrf gm vs k5 (gm)	2011	1515	57	0.038	1722	1309	50	0.038	326	250	12	0.048
nrf gm vs k5 (k5)	2011	1856	428	0.231	1722	1588	387	0.244	326	301	70	0.233
nrf h1 vs k5 (h1)	1098	869	2	0.002	1086	862	2	0.002	95	61	0	0.000
nrf h1 vs k5 (k5)	1098	1010	250	0.248	1086	996	242	0.243	95	84	25	0.298

Caption: The table above shows the number of differential peaks with CNV calls followed by the number differential peaks in CNV regions and the percentage of differential peaks in CNV regions. This data is shown for edgeR with full library size normalization, DiffBind with full library size normalization which is edgeR with background subtracted, and lastly for the peaks that are differential in edgeR but not differential in DiffBind.

REFERENCES

1. Liang K, Keleş S. Normalization of ChIP-seq data with control. BMC Bioinformatics 2012; 13:199

2. Liang K, Keles S. Detecting differential binding of transcription factors with ChIP-seq. Bioinformatics 2012; 28:121–2
